# Association of Relative Pericoronary Adipose Tissue Attenuation with Coronary Artery Calcification Severity

**DOI:** 10.3390/medicina62050990

**Published:** 2026-05-19

**Authors:** Kincső-Zsófia Lőrincz, Raluca Monica Pop, Răzvan-Andrei Licu, Claudia-Raluca Mariean, Andrei Manea, Beáta-Ágota Baróti, Andra-Maria Licu, Fathima Sajeetha Suaibu, Zsuzsánna Pap, Marian Pop

**Affiliations:** 1 Doctoral School of Medicine, George Emil Palade University of Medicine, Pharmacy, Science, and Technology of Targu Mures, 540142 Targu Mures, Romania; 2 Radiology and Medical Imaging Laboratory, Mureș County Emergency Clinical Hospital, 540136 Targu Mures, Romania; 3 Department of Endocrinology, George Emil Palade University of Medicine, Pharmacy, Science, and Technology of Targu Mures, 540142 Targu Mures, Romania; 4 Compartment of Endocrinology, Mures County Clinical Hospital, 540139 Targu Mures, Romania; 5 Department of Pathophysiology, George Emil Palade University of Medicine, Pharmacy, Science, and Technology of Targu Mures, 540142 Targu Mures, Romania; 6 Department of Radiology and Medical Imaging, George Emil Palade University of Medicine, Pharmacy, Science, and Technology of Targu Mures, 540142 Targu Mures, Romania; 7 Radiology and Medical Imaging Laboratory, Covasna County Emergency Hospital “Dr. Fogolyán Kristóf”, 520064 Sfantu Gheorghe, Romania; 8 Department of Anatomy and Embryology, George Emil Palade University of Medicine, Pharmacy, Science, and Technology of Targu Mures, 540142 Targu Mures, Romania

**Keywords:** pericoronary adipose tissue, PCAT attenuation, coronary artery calcium, non-contrast CT, vascular inflammation, imaging biomarkers

## Abstract

*Background and Objectives*: Pericoronary adipose tissue (PCAT) attenuation measured on coronary CT angiography is a promising imaging biomarker of coronary inflammation; however, absolute values may be influenced by technical and inter-individual variability, and a standardized methodology for measurement has not been established. Our study aimed to evaluate the association between PCAT attenuation and CAC burden while comparing absolute attenuation values with normalized values to minimize these sources of variability. *Materials and Methods*: Two hundred patients undergoing cardiac CT were included and stratified into four CAC categories (0, 1–99, 100–299, ≥300). PCAT attenuation was measured at multiple locations on two main levels: aortic root level and four-chamber view level. Relative PCAT attenuation was calculated by subtracting subcutaneous fat attenuation from raw PCAT values. Group comparisons were performed using ANOVA or Kruskal–Wallis tests, and multivariable linear regression models were adjusted for age, sex, and body mass index. *Results*: In univariate analysis, relative PCAT attenuation differed significantly across CAC categories at the aortic-level right coronary artery (RCA) site (*p* = 0.007). In multivariable analysis, higher CAC categories were associated with increased relative PCAT attenuation at the aortic RCA (β = 8.56, *p* = 0.015 for CAC 100–299; β = 10.68, *p* = 0.005 for CAC ≥300), while associations at the left main coronary artery (LMCA) showed significance in low and moderate CAC categories (β = 6.91, *p* = 0.047 for CAC 1–99 and β = 8.57, *p* = 0.016 for CAC 100–299). No significant associations were observed between CAC and raw PCAT attenuation at the aortic level, while isolated and inconsistent findings were observed in other territories. *Conclusions*: Relative PCAT attenuation is independently associated with CAC severity and normalized values may reduce technical and biological variability, potentially enhancing the sensitivity and robustness of this CT-based biomarker.

## 1. Introduction

Cardiovascular disease, including coronary artery disease (CAD), represents a major cause of morbidity and mortality worldwide [1,2,3]. Long-term population studies, such as the Framingham Heart Study, have formed the basis of modern preventive cardiology and identified several key cardiovascular risk factors [4]. Advances in imaging have enabled the development of novel biomarkers, including the non-invasive coronary artery calcium (CAC) score, expanding the focus toward early detection of subclinical disease [5,6,7].

The CAC score directly reflects the present atherosclerotic burden by quantitative measurement of calcified coronary plaques [8,9,10], and is a well-established imaging marker, serving as a “footprint” of chronic disease. However, it lacks the ability to identify the active perivascular inflammation preceding calcium deposition and influencing plaque stability [11].

Recently, cardiovascular research has expanded beyond the vascular wall. Attention has been directed toward epicardial adipose tissue, a unique and metabolically active fat depot, in immediate contact with the myocardium and coronary vessels, with an important role in inflammatory pathways. In advanced coronary artery disease, genes associated with adipocyte browning are downregulated, while those encoding proinflammatory and profibrotic cytokines show increased expression [12]. Epicardial adipose tissue can be assessed using multiple imaging modalities, including echocardiography, cardiac magnetic resonance imaging, and computed tomography, the latter currently representing the reference standard for quantitative pericoronary adipose tissue assessment [13].

Several studies have highlighted the relationship between epicardial fat thickness and inflammatory activity [14,15], associating increased volume with dangerous plaque characteristics, suggesting it independently predicts the risk of myocardial infarction [16]. Emerging evidence suggests that in addition to the macroscopic change, a structural alteration occurs in the pericoronary adipose tissue (PCAT) as a result of paracrine inflammatory signaling from affected coronary vessels [17]. This alteration in the tissue’s composition leads to a measurable change in CT attenuation.

Active perivascular inflammation has been linked to higher attenuation values, resulting from lipid depletion and increased water content [18,19]. Changes in PCAT attenuation have also been shown to respond to medical therapy, such as statin treatment, further supporting its role as a dynamic biomarker of vascular inflammation [20,21].

Current approaches to PCAT evaluation rely on absolute attenuation thresholds, but interpretation is limited by important inter-individual variability, regarding the quantity and quality of systemic adipose tissue, and scanner-related technical factors [22]. Recent work has emphasized the need for standardized approaches to PCAT quantification that account for these factors, ensuring reproducibility and clinical applicability [23,24].

Normalization of PCAT attenuation to subcutaneous fat attenuation may help reduce the influence of systemic adiposity and technical variability by providing an intra-individual reference. We hypothesized that relative PCAT attenuation may better reflect coronary calcification burden than raw attenuation values.

The aim of this study was to assess the association between PCAT attenuation and CAC severity by measuring attenuation at the level of the aortic root and four-chamber view level, and to compare the performance of raw measurements with normalized values, through scaling to subcutaneous adipose tissue.

## 2. Materials and Methods

This retrospective study identified patients who underwent cardiac computed tomography (CT) for suspected CAD, at the Mureș County Emergency Clinical Hospital between November 2021 and October 2022. To reduce selection bias, all consecutive eligible patients during this period were screened for inclusion (*n* = 946). Patients with both zero and non-zero coronary artery calcium (CAC) scores were considered eligible for analysis. Consecutive patient screening continued until each predefined CAC category included 50 eligible patients. Patients were excluded from the study based on the following: missing CT data or poor image quality preventing accurate CAC score or fat attenuation measurement, clinical evidence of active inflammatory heart disease or acute myocardial infarction, history of percutaneous coronary intervention (PCI) or coronary artery bypass grafting (CABG).

**Figure A1:**
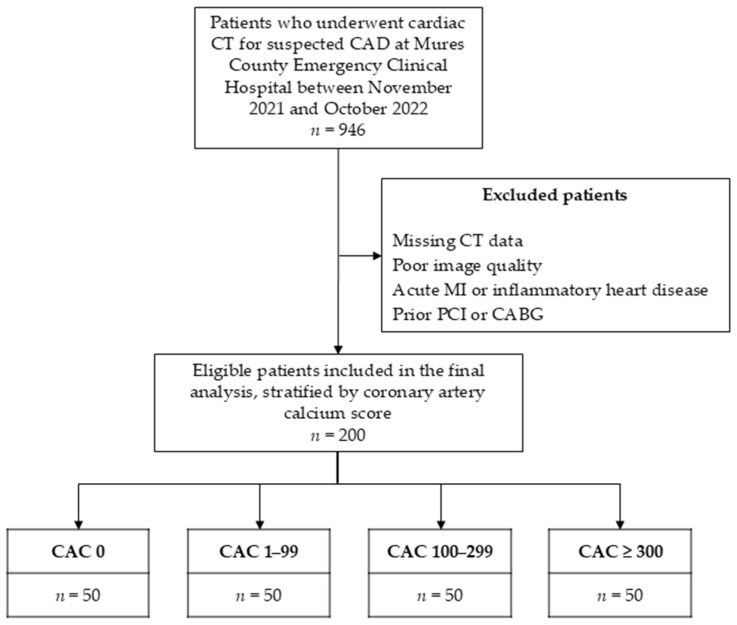
Flowchart of patient selection and study group stratification. Patients who underwent cardiac computed tomography (CT) for suspected coronary artery disease (CAD) were screened for eligibility and categorized according to coronary artery calcium (CAC) score. Patients with both zero and non-zero coronary artery calcium (CAC) scores were considered eligible for analysis. Consecutive patient screening continued until each predefined CAC category included 50 eligible patients.

After applying the exclusion criteria, a final cohort of 200 patients was included in the analysis and stratified according to CAC score into four groups: CAC 0 (*n* = 50), CAC 1–99 (*n* = 50), CAC 100–299 (*n* = 50), and CAC ≥300 (*n* = 50). The equal group distribution was performed to allow balanced comparative analysis across different degrees of coronary calcification. The study flowchart is summarized in Figure A1.

The study was conducted in accordance with the Declaration of Helsinki. Patient demographic data, including biological sex, date of birth, and age at the time of imaging, were extracted from the national identification number (CNP). Additional anthropometric data were collected at the time of the CT scan, specifically height and weight, to calculate body mass index (BMI). All data were anonymized to ensure privacy.

### 2.1. Imaging Protocol and Measurements

A standard non-contrast CT SmartScore acquisition protocol was used for all scans on a GE Revolution HD scanner (GE HealthCare, Milwaukee, WI, USA) (120 kV tube voltage, 250 mA tube current, 25 cm field of view and 2.5 mm slice spacing).

#### 2.1.1. Coronary Artery Calcium Score

CAC scores were measured using the Agatston method, with the cvi42 dedicated software (version 5.13.9, Circle Cardiovascular Imaging Inc., Calgary, AB, Canada). The analysis included four primary vessels: right coronary artery (RCA), the left main coronary artery (LMCA), left anterior descending artery (LAD) and left circumflex artery (LCx).

#### 2.1.2. Pericoronary Adipose Tissue Attenuation

Attenuation analysis was performed using RadiAnt DICOM Viewer (version 2020.2, Medixant, Poznań, Poland). Fat attenuation was sampled manually, in 2D elliptical regions of interest (ROI), expressed in HU (Hounsfield Units), in the standard mediastinal window settings (Width: 400 HU; Level: 40 HU), on two standardized anatomical levels:

- Aortic root level: ROIs were placed adjacent to the RCA and the LMCA;
- Four-chamber view level: ROIs were placed adjacent to the RCA, LCx and LAD.

To ensure measurement consistency, each PCAT ROI maintained a minimum area of 0.1 cm^2^, positioned within a radial distance from the target vessel wall approximately equal to the diameter of the adjacent vessel.

#### 2.1.3. Subcutaneous Fat Attenuation

To distinguish the localized inflammatory effects of coronary atherosclerosis from those of generalized systemic adiposity, subcutaneous fat (SF) was sampled as an internal control. ROIs with a minimum area of 1.0 cm^2^ were placed within the anterior thoracic subcutaneous tissue at the same two axial levels as the cardiac measurements.

**Figure 1:**
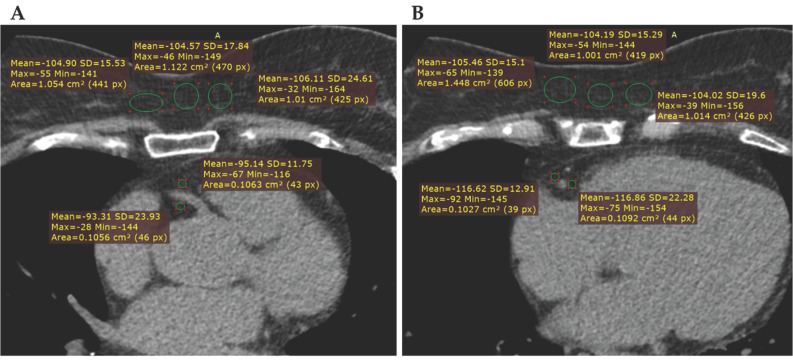
Showing two axial non-enhanced CT images, with examples for ROI placement (green circles) in the RCA pericoronary adipose tissue and subcutaneous adipose tissue at the aortic root level (**A**) and the four-chamber view level (**B**). The adjacent yellow annotations show the ROI-derived attenuation parameters, including mean attenuation in Hounsfield units (HU), standard deviation (SD), maximum and minimum HU values, and ROI area in cm^2^/pixels.

Examples for ROI placement at the aortic root level and four-chamber view level are demonstrated in Figure 1.

### 2.2. Statistical Analysis

Statistical analysis was performed using Microsoft Excel (version 16.0, Microsoft Corp., Redmond, WA, USA), and JASP (version 0.16.4, JASP Team, Amsterdam, The Netherlands).

#### 2.2.1. Descriptive Statistics

Continuous variables, including pericoronary and subcutaneous adipose tissue attenuation, age and body mass index, were summarized as mean ± standard deviation (SD) or median and interquartile range (IQR), as appropriate. CAC scores were categorized into clinically relevant groups for analysis: CAC 0 (non-calcified), CAC 1–99 (mild), CAC 100–299 (moderate), CAC ≥300 (severe).

Pericoronary fat attenuation (HU_PCAT_) was then normalized to subcutaneous fat attenuation (HU_SF_) to provide a relative value (hereafter referred to as relative PCAT attenuation) for analysis, using the following formula:

HU_RelativePCAT_ = HU_PCAT_ − HU_SF_

#### 2.2.2. Inferential Statistics

Data normality was rigorously assessed using the Shapiro–Wilk test, and homogeneity of variance was evaluated using Levene’s test to confirm the appropriateness of parametric modeling. Differences in PCAT attenuation across CAC categories with normal distributions and equal variances were evaluated using one-way ANOVA, followed by Tukey HSD post hoc testing for multiple comparisons. When homogeneity of variance was violated, Welch’s ANOVA was applied. For non-normally distributed variables, including age and BMI, comparisons were performed using the Kruskal–Wallis H test. Sex was evaluated as a categorical variable using chi-square analysis.

Multivariable linear regression was utilized to determine the independent association between CAC severity and PCAT attenuation, with models adjusted for age, sex, and BMI. CAC = 0 was used as the reference category. β coefficients represent the change in attenuation, expressed in Hounsfield units (HU).

Primary analyses were performed on the full dataset. Sensitivity analyses excluding outliers confirmed the robustness of the findings.

All statistical tests were two-tailed, and a *p*-value of <0.05 was considered statistically significant.

## 3. Results

### 3.1. Study Population

A total of 200 patients (mean age 58.3 ± 10.5 years, 53.5% male) were included in the analysis, evenly distributed across CAC categories (*n* = 50 for each group). Baseline characteristics are summarized in Table 1. Increasing CAC severity was associated with older age and a higher proportion of male patients. Median age increased progressively from 50.5 years in the CAC 0 group to 64.0 years in the CAC ≥300 group (*p* < 0.001). Similarly, the proportion of male patients was significantly higher in advanced CAC groups (64% in CAC ≥ 100 vs. 42–44% in lower CAC groups; *p* = 0.031). No significant differences in BMI were observed across CAC categories.

### 3.2. Group Comparisons of Attenuation

A comparative analysis of raw and relative PCAT attenuation across CAC severity groups through univariate ANOVA with post hoc comparisons, is summarized in Table 2.

#### 3.2.1. Relative PCAT Attenuation

**Figure 2:**
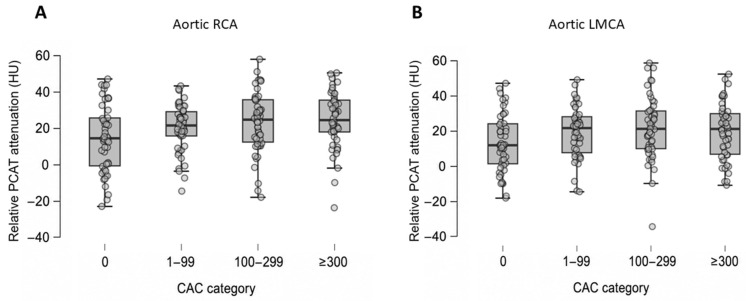
Relative PCAT attenuation across coronary artery calcium (CAC) severity categories at the aortic level. Panel (**A**) displays the distribution of relative PCAT attenuation at the level of the right coronary artery, and panel (**B**) displays attenuation at the level of the left main coronary artery. Horizontal lines indicate the median, boxes represent the interquartile range (IQR), and whiskers extend to 1.5× IQR. A progressive increase in relative PCAT attenuation is observed with increasing CAC severity, particularly at the Ao RCA. PCAT: pericoronary adipose tissue; HU: Hounsfield Units; CAC: coronary artery calcium; RCA: Right coronary artery; LMCA: Left main coronary artery.

Relative PCAT attenuation differed across CAC groups at the aortic level (Figure 2).

Relative PCAT values differed significantly across CAC categories for aortic RCA (*p* = 0.007). Mean aortic RCA attenuation showed a progressive increase from CAC 0 to higher CAC groups (CAC 0: 13.45 ± 18.50 HU vs. CAC ≥300: 24.13 ± 16.33 HU). Post hoc analysis using Tukey’s test revealed a significant difference between CAC 0 and CAC 100–299, as well as CAC 0 and CAC ≥300.

For aortic LMCA, mean relative PCAT values differed across CAC categories with borderline significance (*p* = 0.050), without a consistent dose–response pattern.

At the four-chamber level, no statistically significant differences were observed across CAC groups in univariate analysis, for any coronary territory.

#### 3.2.2. Raw PCAT Attenuation

In contrast, raw PCAT attenuation did not show significant differences across CAC categories at any of the measurement sites.

### 3.3. Multivariable Association of CAC Severity with PCAT Attenuation

To determine the independent relationship between CAC severity and PCAT attenuation, multivariable linear regression was performed, adjusting for age, sex, and BMI.

#### 3.3.1. Relative PCAT

**Figure 3:**
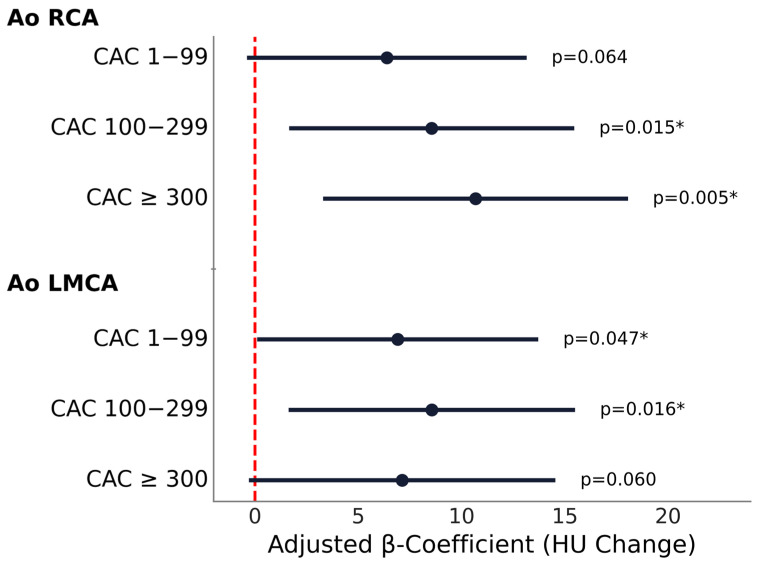
Association between relative PCAT attenuation and CAC severity across measurement sites. Forest plot showing multivariable regression results for relative PCAT attenuation at the aortic level for RCA and LMCA. Black dots represent adjusted β-coefficient estimates and error bars represent 95% confidence intervals. The vertical dashed line at 0 indicates no effect. Statistically significant *p* values (*p* < 0.05) are indicated by an asterisk (*). HU: Hounsfield unit; Ao: aortic level; RCA: right coronary artery; LMCA: left main coronary artery.

Relative PCAT attenuation increased significantly with greater CAC burden at the aortic level, and to a lesser extent at the 4-chamber level, with a progressive increase in attenuation observed for the aortic RCA and a less consistent pattern for the aortic LMCA (Figure 3).

At the aortic RCA, higher CAC categories were associated with significantly higher relative PCAT attenuation, when compared with CAC 0, with β coefficients of 8.56 (95% CI: 1.66 to 15.46, *p* = 0.015) for CAC 100–299 and 10.68 (95% CI: 3.30 to 18.06, *p* = 0.005) for CAC ≥300. CAC 1–99 showed a non-significant trend (*p* = 0.064). Sensitivity analysis excluding outliers demonstrated consistent results, with similar effect sizes and persistent significance for both moderate and high CAC categories.

For the aortic LMCA, CAC 1–99 (β = 6.91, 95% CI 0.11 to 13.72, *p* = 0.047) and CAC 100–299 (β = 8.57, 95% CI 1.63 to 15.50, *p* = 0.016) were significantly associated with higher relative PCAT attenuation values, while CAC ≥300 group reached only borderline association (β = 7.13, 95% CI −0.29 to 14.55, *p* = 0.060). Sensitivity analysis excluding outliers demonstrated consistent results, the association between relative PCAT attenuation and CAC remained significant across the low and moderate CAC categories, with similar direction and magnitude of effect.

Multivariable regression results for other coronary segments are provided in Table A1. In the 4-chamber view, relative PCAT attenuation demonstrated heterogeneous associations across coronary territories. At the RCA, a significant association was observed only for CAC ≥300 (β = 10.07, 95% CI 2.41 to 17.73, *p* = 0.010), whereas lower CAC categories were not significant. At the LAD, both CAC 100–299 (β = 7.14, 95% CI 1.13 to 13.15, *p* = 0.020) and CAC ≥300 (β = 7.17, 95% CI 0.75 to 13.60, *p* = 0.029) were independently associated with higher attenuation. No significant associations were observed for the LCx.

Among covariates, age was independently associated with PCAT attenuation at the LAD (β = −0.27, 95% CI −0.48 to −0.05, *p* = 0.016), while sex and BMI were not significantly associated in any model.

#### 3.3.2. Raw PCAT Attenuation

Multivariable regression analyses using raw PCAT values at the aortic and 4-chamber levels are provided in Table A2 and Table A3.

At the aortic level, no significant associations were observed between CAC categories and PCAT attenuation at either the RCA or LMCA, although a non-significant trend toward higher attenuation was observed at the RCA in the CAC ≥300 group. At this level, sex was independently associated with higher PCAT attenuation at both the RCA and LMCA, while no associations were observed for age or BMI.

At the 4-chamber level, no significant associations were observed for the RCA or LAD across CAC categories. In contrast, the LCx demonstrated significant negative associations for CAC 1–99 (β = −7.15, 95% CI −12.59 to −1.71, *p* = 0.010) and CAC ≥300 (β = −6.78, 95% CI −12.71 to −0.84, *p* = 0.025), while no association was observed for CAC 100–299, resulting in an inconsistent pattern across CAC categories.

## 4. Discussion

Our findings demonstrated that pericoronary adipose tissue attenuation, when normalized to subcutaneous fat, is independently associated with coronary artery calcification burden, especially at the aortic root level around the right coronary artery. The results remained significant after adjusting for age, sex, and body mass index, suggesting that relative PCAT attenuation is likely influenced by the inflammatory status of the neighboring vessel and is not solely a reflection of systemic adiposity. In contrast, raw attenuation values did not demonstrate a consistent association with CAC burden across measurement sites. As expected, increasing CAC burden was associated with older age and male sex, supporting the validity of the study population.

### 4.1. Imaging Biomarkers of Coronary Plaques

Several imaging biomarkers have been established for coronary plaque evaluation, including anatomical (quantity and distribution), morphological (plaque attenuation, vascular remodeling), and hemodynamic (fractional flow reserve) [25,26]. Identifying molecular and inflammatory biomarkers with PET CT can add a new depth to risk stratification; however, it implies a less accessible and more complex imaging protocol [27].

Recently, attention has shifted toward the perivascular microenvironment. While earlier studies focused on the relationship between epicardial adipose tissue volume and coronary atherosclerosis, emerging evidence suggests that pericoronary fat attenuation is more strongly associated with the pathology [28]. Pericoronary adipose tissue attenuation measurement has emerged as a promising tool for detecting local inflammation, serving as a non-invasive imaging biomarker readily available on conventional CT imaging.

### 4.2. PCAT as a Surrogate for Inflammation

This approach is based on the concept that the coronary vessel wall and surrounding adipose tissue engage in bidirectional signaling; thus, inflammatory changes within the vessel could be reflected in the surrounding fat. A biopsy-proven study by Antonopoulos et al. concluded that active inflammation leads to a higher PCAT attenuation by inhibiting preadipocyte differentiation through paracrine signaling and cytokine accumulation [18].

Although our study did not assess individual plaque characteristics, our findings are consistent with prior studies [20,29,30], indicating that increased PCAT attenuation is associated with atherosclerotic disease.

### 4.3. Relationship Between PCAT and CAC

The CAC score is a cornerstone of cardiovascular risk stratification and reflects cumulative atherosclerotic burden. However, extensive calcification is known to represent stable plaques, rather than the active inflammation that characterizes higher-risk, unstable plaques [29,31]. PCAT attenuation is an emerging biomarker that aims to identify the active inflammatory shift in the perivascular environment.

Our study observed an increase in relative PCAT attenuation with higher CAC burden, possibly reflecting a state of residual inflammatory activity in the presence of calcified plaques. This pattern was non-linear, with more pronounced differences at higher CAC levels.

The strongest and most consistent association of increasing attenuation with higher CAC burden was identified at the aortic root level of the right coronary artery. In our multivariable analysis performed at this site, the highest CAC group demonstrated a β of 10.68 HU. The association remained consistent after removing outliers, with similar direction and magnitude of effect. This increase in attenuation likely reflects the ‘aqueous shift’, indicating transition of perivascular fat from a lipid-rich to a water-rich state.

Similarly, previous studies have concluded that the RCA was the most reproducible and representative site for PCAT attenuation analysis [18,30,32]. The CRISP-CT trial has demonstrated that PCAT attenuation, when measured around the proximal segment of the RCA, is a robust predictor of adverse cardiovascular outcomes and outperforms measurements taken in other coronary segments. This may be related to reduced motion artifacts and more consistent anatomical sampling at this location [33].

At the left main coronary artery, our initial univariate analysis (ANOVA) did not identify a significant association with CAC burden. However, in the multivariate regression model, after adjusting for age, sex, and BMI, it differed significantly across low and moderate CAC categories, when compared to CAC 0, while the highest CAC group showed borderline significance. This change could suggest that this relationship may have been masked by demographic and systemic confounders.

At the four-chamber view level, our study observed that the association between CAC burden and relative PCAT attenuation was less consistent across territories compared to the aortic level. While no differences were observed in univariate analysis, the RCA and LAD demonstrated independent associations with high CAC burden (CAC ≥300) in multivariate analysis, suggesting that the local inflammatory signal could be sensitive to demographic factors. Findings for the LCx were not consistent across analyses, including paradoxical negative associations. Intra-individual variation is in line with prior results in stable CAD patients, which have demonstrated minor, but significant differences in attenuation between the LAD, LCx, and RCA [34]. These findings suggest that coronary inflammation may be heterogeneous across different regions and support the growing consensus that PCAT attenuation may not be reliably extrapolated from a single artery or a single measurement site.

While PCAT attenuation changes have been consistently linked to CAD, its relationship with CAC is not universally observed in the literature. In the CRISP- CT cohort, the pericoronary attenuation, quantified as the fat attenuation index (FAI), was not associated with either segmental or total coronary calcium score [33]. The study by Antonopoulos et al. found a weak association between FAI in the PCAT and total CAC [18]. Furthermore, Liu et al. reported a very small association in multivariate analysis between CAC and PCAT (β = 0.002) [35].

These inconsistencies highlight the methodological challenges in PCAT quantification and point to the need for standardized approaches that reduce variability, while preserving biologically meaningful signals.

### 4.4. Importance of Relative PCAT Attenuation

Large-scale trials have proposed PCAT attenuation cut-off values for cardiovascular risk stratification [26,33]. Values ranging from −70 HU [33] to −77 HU [36], measured at the proximal RCA, point to a significantly higher risk of major adverse cardiovascular event (MACE). However, the optimal method for PCAT quantification has yet to be fully established.

Differences in CT acquisition parameters, particularly tube voltage, have been shown to significantly alter attenuation values [37,38]. In addition, patient-specific factors further influence attenuation measurements [39,40] and PCAT attenuation may vary according to sex and coronary vessel [22]. In patients with severe CAC, PCAT measurements should be interpreted cautiously, as extensive calcification may introduce blooming artifacts and reduce the precision of perivascular fat delineation. Moreover, in coronary CT angiography, the intraluminal attenuation contributes to variations in adjacent fat measurements, introducing an additional source of variability [39,41].

In the original PCAT methodology, “adjusted attenuation” addresses anatomical differences by measuring attenuation in a specific region within a radial extent equal to the local vessel diameter, ensuring standardization across vessels of different sizes [18]. Some studies explored an adjusted perivascular attenuation score by weighting for technical parameters and anatomical factors related to the fat distribution around the coronary arteries [23,33], or by dividing mean PCAT attenuation by the corresponding vessel-specific mean lumen attenuation [42].

Normalization principles are also applied in studies using PET-CT imaging, where the target to background ratio (TBR) is calculated, to stabilize metabolic measurements against a blood pool reference [18]. To isolate local inflammatory gradients Antonopoulos et al. proposed the Volumetric Perivascular Characterization Index (VPCI) to measure the percentage change in attenuation between PCAT and fat located 20 mm away (considered non-coronary fat), and found a weak association between PCAT and the segmental CAC at the RCA, but not with total calcium burden [18]. Moreover, to this date no clear biological definition of PCAT exists.

Building on prior evidence that PCAT reflects local vascular inflammation independently of systemic adiposity [30], the use of subcutaneous fat as an internal reference may further enhance measurement stability.

Subcutaneous adipose tissue and PCAT differ in embryological origin, anatomical location, and metabolic activity, and subcutaneous fat may itself be influenced by systemic metabolic and pharmacological factors. Nevertheless, we selected subcutaneous fat as an intra-individual reference to reduce inter-patient and technical variability affecting absolute attenuation values, rather than to imply biological equivalence between the two adipose compartments.

In our study, raw PCAT attenuation was scaled to subcutaneous fat attenuation, and this relative PCAT value demonstrated positive associations with coronary artery calcification. In contrast, raw PCAT attenuation did not have a consistent independent association with CAC across measurement sites. This discrepancy suggests that, without subcutaneous fat-based normalization, the extent of the perivascular inflammatory changes may be underestimated. However, in the four-chamber view of the LCx, raw values demonstrated a paradoxical negative association with CAC burden, without a consistent dose–response relationship. This result suggests that measurements in certain territories could be more susceptible to local anatomical variability, possibly attributable to narrow pericoronary space and smaller vessel calibers.

Notably, as our analysis was performed on non-contrast CT images, the potential confounding effect of intraluminal contrast attenuation was eliminated. This approach enables the assessment of coronary inflammation during routine calcium scoring scans, potentially supporting the application of inflammatory biomarkers to a broader population, without the risks or costs associated with contrast-enhanced scans.

In the literature, we found that in non-cardiac CT applications, namely in the inflammatory bowel disease setting, perilesional fat attenuation measurement has been interpreted relative to subcutaneous tissue as an internal reference [43]. To our knowledge, this is among the first studies to apply subcutaneous fat-based normalization of PCAT attenuation in cardiac CT, where absolute attenuation thresholds remain the dominant methodology.

### 4.5. Study Limitations

Limitations of our study include a retrospective design, a relatively small sample size from a single institution, and use of a CT scanner from a single vendor. Comprehensive clinical data regarding cardiovascular risk factors and medical therapy, including hypertension, diabetes mellitus, smoking status, hypercholesterolemia, and statin use, were not consistently available for all patients due to the retrospective design and outpatient-based data collection, and therefore could not be included in the multivariable analysis. Consequently, residual confounding cannot be excluded. The relatively small single-center cohort and the variability of PCAT attenuation values may have limited the ability to detect smaller intergroup differences. Additionally, the clinical utility of pericoronary fat attenuation remains unestablished in non-contrast CT [44]. It should also be noted that while pericoronary attenuation was normalized to subcutaneous fat, the anatomical differences between the two may not fully mitigate all potential confounders, as subcutaneous adipose tissue may itself be influenced by systemic metabolic conditions and pharmacological treatment.

Furthermore, extensive coronary calcification may reduce the reproducibility of PCAT attenuation measurements, particularly in heavily calcified vessels where blooming artifacts and impaired delineation of the vessel wall could influence ROI placement and adjacent fat attenuation assessment.

Because ROI placement was performed manually, the absence of dedicated intra- and inter-observer reproducibility assessment represents an additional limitation, and potential observer-related variability cannot be fully excluded.

## 5. Conclusions

Our study concluded that pericoronary fat attenuation, when normalized by scaling against subcutaneous fat attenuation, is independently associated with coronary artery calcification severity. Relative attenuation values showed a significant positive association with CAC, whereas raw attenuation values failed to provide significant or consistent relationships.

The right coronary artery at the aortic level proved to be the most reliable site for assessment, showing the most pronounced associations. Our findings suggest that normalized values may contribute to reducing technical and biological variability, potentially enhancing the sensitivity and robustness of this CT-based biomarker. Furthermore, the use of non-contrast CT increases the feasibility of incorporating this approach into routine calcium scoring examinations.

Further validation in larger, multi-center populations across diverse hardware and scanning parameters is required to establish a standardized method for PCAT measurement and interpretation.

## Figures and Tables

**Table 1:** Baseline demographic and clinical characteristics across CAC groups.

Variable	CAC 0	CAC 1–99	CAC 100–299	CAC ≥300	*p*-Value
Age (years)	50.5 [42.2 to 59.0]	59.0 [54.0 to 67.8]	59.5 [53.0 to 65.0]	64.0 [59.5 to 71.0]	**<** **0** **.001**
Sex (male), *n* (%)	21 (42%)	22 (44%)	32 (64%)	32 (64%)	**0.031**
BMI (kg/m^2^)	27.7 [24.6 to 30.9]	30.2 [27.2 to 31.7]	29.2 [27.1 to 32.1]	29.7 [26.8 to 33.7]	0.110

Values are presented as median [interquartile range] for continuous variables and counts (percentages) for categorical variables. Comparisons across CAC groups were performed using the Kruskal–Wallis H test for continuous variables and the chi-square test for categorical variables. Statistically significant values (*p* < 0.05) are shown in bold. BMI: body mass index.

**Table 2:** Pericoronary fat attenuation across CAC groups.

Measurement Site	CAC 0	CAC 1–99	CAC 100–299	CAC ≥300	*p*-Value	Post hoc
Relative PCAT (HU)						
Aortic RCA	13.45 ± 18.50	19.24 ± 12.99	22.38 ± 16.78	24.13 ± 16.33	**0.007**	0 vs. 100–299 0 vs. ≥300
Aortic LMCA	13.63 ± 16.45	19.85 ± 14.57	22.24 ± 17.89	20.40 ± 16.00	**0.050**	—
4C RCA	16.36 ± 17.27	19.50 ± 15.19	18.34 ± 17.61	23.34 ± 17.18	0.211	—
4C LCx	17.55 ± 15.76	14.78 ± 13.99	18.66 ± 15.76	16.80 ± 15.50	0.634	—
4C LAD	21.46 ± 12.54	23.79 ± 13.77	25.16 ± 14.83	23.79 ± 15.85	0.630	—
Raw PCAT (HU)						
Aortic RCA	−104.0 ± 17.03	−101.0 ± 9.88	−98.18 ± 13.65	−98.43 ± 12.69	0.190	—
Aortic LMCA	−103.84 ± 14.44	−100.40 ± 11.80	−98.32 ± 14.34	−102.16 ± 12.84	0.200	—
4C RCA	−101.06 ± 15.58	−101.22 ± 13.15	−102.70 ± 14.99	−98.17 ± 14.16	0.466	—
4C LCx	−99.88 ± 13.48	−105.93 ± 11.54	−102.38 ± 13.81	−104.71 ± 12.86	0.097	—
4C LAD	−95.97 ± 11.08	−96.93 ± 14.61	−95.88 ± 13.17	−97.71 ± 14.22	0.888	—

Data are presented as mean ± standard deviation. Group comparisons were performed using one-way ANOVA. When homogeneity of variance was violated, Welch’s ANOVA was applied. Statistically significant values (*p* < 0.05) are shown in bold. PCAT: pericoronary adipose tissue; CAC: coronary artery calcium; HU: Hounsfield Units; 4C: 4 chamber level; RCA: right coronary artery; LMCA: left main coronary artery; LCx: left circumflex artery; LAD: left anterior descending artery.

## Data Availability

Data are contained within the article.

## Abbreviations

The following abbreviations are used in this manuscript:

CAD	Coronary artery disease
CAC	Coronary artery calcium
PCAT	Pericoronary adipose tissue
CT	Computed tomography
PCI	Percutaneous coronary intervention
CABG	Coronary artery bypass grafting
BMI	Body mass index
RCA	Right coronary artery
LMCA	Left main coronary artery
LAD	Left anterior descending artery
LCx	Left circumflex artery
ROI	Region of interest
HU	Hounsfield units
SF	Subcutaneous fat

## Appendix A

**Table A1:** Multivariable regression analysis of relative 4-chamber PCAT values.

Variable	4C RCA β (95% CI)	*p*-Value	4C LCx β (95% CI)	*p*-Value	4C LAD β (95% CI)	*p*-Value
CAC 1–99	5.26 (−1.77 to 12.28)	0.141	−3.71 (−10.14 to 2.72)	0.256	5.21 (−0.68 to 11.11)	0.083
CAC 100–299	4.07 (−3.09 to 11.23)	0.263	0.33 (−6.23 to 6.88)	0.922	**7.14 (1.13 to 13.15)**	**0.020**
CAC ≥300	**10.07 (2.41 to 17.73)**	**0.010**	−2.04 (−9.05 to 4.97)	0.567	**7.17 (0.75 to 13.60)**	**0.029**
Sex	−0.97 (−5.89 to 3.95)	0.699	−0.92 (−5.42 to 3.58)	0.686	−3.31 (−7.44 to 0.82)	0.115
Age	−0.15 (−0.41 to 0.10)	0.243	0.11 (−0.12 to 0.35)	0.351	**−0.27 (−0.48 to −0.05)**	**0.016**
BMI	−0.43 (−0.89 to 0.04)	0.070	0.01 (−0.41 to 0.43)	0.955	−0.30 (−0.69 to 0.09)	0.127

Reference category = CAC 0. Models adjusted for age, sex, and BMI. Statistically significant values (*p* < 0.05) are shown in bold. RCA: right coronary artery; LCx: left circumflex artery; LAD: left anterior descending artery.

**Table A2:** Multivariable regression analysis of raw (mean) aortic PCAT values.

Variable	Ao RCA β (95% CI)	*p*-Value	Ao LMCA β (95% CI)	*p*-Value
CAC 1–99	3.59 (−2.01 to 9.18)	0.207	4.11 (−1.45 to 9.68)	0.147
CAC 100–299	5.22 (−0.49 to 10.92)	0.073	5.22 (−0.45 to 10.90)	0.071
CAC ≥300	5.29 (−0.81 to 11.39)	0.089	1.74 (−4.33 to 7.81)	0.572
Sex	**5.50 (1.58 to 9.41)**	**0.006**	**4.33 (0.44 to 8.23)**	**0.029**
Age	−0.04 (−0.25 to 0.16)	0.685	−0.05 (−0.25 to 0.15)	0.640
BMI	−0.17 (−0.54 to 0.19)	0.351	−0.18 (−0.55 to 0.18)	0.321

Reference category = CAC 0. Models adjusted for age, sex, and BMI. Statistically significant values (*p* < 0.05) are shown in bold. Ao: aortic level; RCA: right coronary artery; LMCA: left main coronary artery; BMI: body mass index.

**Table A3:** Multivariable regression analysis of raw (mean) 4-chamber PCAT values.

Variable	4C RCA β (95% CI)	*p*-Value	4C LCx β (95% CI)	*p*-Value	4C LAD β (95% CI)	*p*-Value
CAC 1–99	1.82 (−4.21 to 7.85)	0.552	**−7.15 (−12.59 to −1.71)**	**0.010**	1.77 (−3.74 to 7.29)	0.526
CAC 100–299	−0.17 (−6.32 to 5.98)	0.957	−3.91 (−9.46 to 1.64)	0.166	2.90 (−2.72 to 8.52)	0.310
CAC ≥300	5.33 (−1.25 to 11.90)	0.112	**−6.78 (−12.71 to −0.84)**	**0.025**	2.43 (−3.58 to 8.44)	0.426
SEX	1.61 (−2.61 to 5.83)	0.452	1.66 (−2.15 to 5.47)	0.392	−0.73 (−4.59 to 3.13)	0.710
AGE	−0.16 (−0.38 to 0.06)	0.164	0.11 (−0.09 to 0.31)	0.286	**−0.27 (−0.47 to −0.07)**	**0.009**
BMI	−0.36 (−0.75 to 0.04)	0.074	0.08 (−0.28 to 0.43)	0.670	−0.24 (−0.60 to 0.13)	0.202

Reference category = CAC 0. Models adjusted for age, sex, and BMI. Statistically significant values (*p* < 0.05) are shown in bold. RCA: right coronary artery; LCx: left circumflex artery; LAD: left anterior descending artery; BMI: body mass index.
